# The Prevalence of Reduced Bone Mineral Density and the Impact of Specific Auxological Factors and Hormones on Bone Mass in Children with Endocrine Disorders

**DOI:** 10.3390/jcm14092988

**Published:** 2025-04-25

**Authors:** Anna Łupińska, Sara Aszkiełowicz, Arkadiusz Zygmunt, Andrzej Lewiński, Renata Stawerska

**Affiliations:** 1 Department of Endocrinology and Metabolic Diseases, Polish Mother’s Memorial Hospital Research Institute, 93-338 Lodz, Poland; sara.aszkielowicz@iczmp.edu.pl (S.A.); arkadiusz.zygmunt@umed.lodz.pl (A.Z.); renata.stawerska@icloud.com (R.S.); 2 Department of Pediatric Endocrinology, Medical University of Lodz, 93-338 Lodz, Poland; 3 Department of Endocrinology and Metabolic Diseases, Medical University of Lodz, 93-338 Lodz, Poland

**Keywords:** bone mass, hormones, children, IGF-1, dual X-ray densitometry, bone mineral density

## Abstract

**Background/Objectives**: The skeletal system reaches peak bone mass through modeling and remodeling processes, influenced by environmental, dietary, hormonal, and genetic factors. In children with endocrinopathies, disturbances in bone mass and mineralization may correlate with hormonal levels, but conditions like short stature or obesity can confound DXA results. This study aimed to assess the prevalence of decreased bone mineral density (BMD) in children with endocrine disorders and evaluate the impact of auxological and hormonal abnormalities on BMD. **Methods:** This study analyzed medical records of 148 children (mean age 11.85 ± 3.34 years); 73 girls and 75 boys). Conditions included obesity (22.9%), short stature (47.9%), precocious puberty (10.1%), and other diagnoses. Clinical data included primary diagnosis, height, body weight, pubertal stage, and serum concentrations of calcium, phosphate, alkaline phosphatase, 25OHD, PTH, osteocalcin, Crosslaps, TSH, fT4, IGF-1, IGF-BP3, cortisol, estradiol, testosterone, and bone age. DXA scans were performed at the total body less head (TBLH) and lumbar spine (Spine) projection. **Results:** Low bone mass (aBMD Z-score ≤ −2) was found in 34.46% at TBLH and 15.54% at the Spine. After height adjustment (HAZ adjustment), the prevalence of low bone mass decreased to 11.4% at TBLH and 4.1% at the Spine. In the short stature group, the normalization of Z-scores for height significantly reduced abnormal results. A positive correlation was found between DXA parameters and age, height standard deviation score (HSDS), BMI SDS, estradiol, testosterone, IGF-1, and IGF-BP3. A negative correlation existed between vitamin D and DXA parameters. Bone turnover markers (osteocalcin and Crosslaps) also negatively affected bone mass. No significant correlations were found with PTH, TSH, fT4, or cortisol. In children with growth retardation, lower aBMD_HAZ_ Z-scores were observed in those with decreased IGF-1. Positive correlations existed between BMI SDS, IGF-1, and adjusted aBMD Z-scores. **Conclusions:** Children with endocrine disorders, especially those with short stature, are at risk for bone mineralization disorders. Height normalization is crucial for accurate DXA interpretation and avoiding overdiagnosis. Positive influences on bone mass include height, BMI, IGF-1, estradiol, and testosterone, while negative factors include bone turnover markers and low vitamin D.

## 1. Introduction

The skeleton undergoes a significant transformation throughout typical childhood and adolescence. Through modeling and remodeling, the skeletal system achieves its adult configuration and ultimately reaches peak bone mass, defined as the maximum bone mineral density (BMD) attained by approximately 30 years of age [1]. At this stage, bones attain their highest strength and density.

The optimization of peak bone mass requires a complex interplay of environmental, dietary, hormonal, and genetic factors. A range of chronic conditions, as well as genetic polymorphisms, are associated with a reduced bone density, potentially increasing the risk of fractures both during childhood and later in adulthood. Notably, approximately 90% of the total bone mass is accrued by the end of the second decade of life, with about 50% of the skeletal mass formed during adolescence [2].

The peak bone mass achieved during puberty plays a more critical role in the pathogenesis of osteoporosis than the subsequent loss of bone mass that occurs later in life [3].

Dual-energy X-ray absorptiometry (DXA) is the gold standard for evaluating BMD. This method utilizes low-and high-energy X-rays to simultaneously assess BMD and soft tissue composition, differentiating between fat mass (FM) and lean body mass (LBM). DXA allows for measuring the absolute amount of bone mineral content (BMC) expressed in grams and its projected measurement area (BA) expressed in cm^2^. The results of DXA are reported as areal bone mineral density (aBMD), which is the ratio of BMC to BA. Thus, BMD is expressed in g/cm^2^ rather than g/cm^3^, as the term “density” might suggest.

The posterior–anterior spine and total body less head (TBLH), encompassing all bones of the skeleton except the cranial and facial bones, are the preferred and recommended skeletal sites for BMC and BMD measurements in pediatric patients, as endorsed by the International Society for Clinical Densitometry (ISCD) [4]. One limitation of the DXA method, based on the attenuation of X-ray beams passing through the measured body part, is that the results are influenced by the bone size and the degree of X-ray beam penetration to the detector. Consequently, this measurement may be affected by the structural dimensions of the bones being assessed [5,6]. The current standard for reporting DXA results in pediatrics is the BMD Z-score, which reflects the number of standard deviations (SD) above or below the mean for chronological age and sex. A Z-score of ≤−2.0 SD is classified as “low bone mass or low bone density” [4,7].

Previously, some clinicians and researchers used the term “osteopenia” in children with Z-scores below −1.0 SD [6]. However, this terminology is no longer applied to the pediatric population. It is now suggested that a Z-score between −1.0 SD and +1.0 SD corresponds to normal bone density, while values below −1.0 SD warrant careful clinical monitoring.

BMD in DXA assessments is highly size-dependent and influenced by growth. Changes in bone size during childhood complicate the interpretation of DXA results [6]. Consequently, BMD is not regarded as a sensitive measure of bone acquisition in childhood because of the continuous changes in bone geometry and the inability to distinguish changes resulting from growth versus mineralization [8]. This represents one of the major limitations of DXA in pediatric evaluations. Moreover, BMD measurements may be underestimated in children who are short for their age and overestimated in those who are tall. An abnormal BMD Z-score should prompt a consideration of confounding factors such as height, before diagnosing low bone density, as the bone surface area does not accurately reflect true bone size [6]. Moreover, factors that can also influence BMD include body mass and pubertal stage.

No clear consensus exists regarding adjustments for BMC or BMD in children with short or tall stature [9,10]. Recently, the ISCD convened a panel to address the clinical application of DXA in children, recommending that in cases of short stature or growth delay, BMC and aBMD results for the Spine and TBLH should be adjusted. Adjustments can be made for the Spine using bone mineral apparent density (BMAD) or the height Z-score. For TBLH, adjustments should use the height Z-score [4,10]. The most commonly employed method for size adjustment in pediatric DXA is the calculation of BMAD, which estimates volumetric bone density [11]. Total body measurements primarily reflect skeletal growth and mineralization rather than serving as direct predictors of fracture risk.

Metabolic changes occurring in a growing body are also reflected in bone turnover markers, including markers of bone formation (e.g., alkaline phosphatase, osteocalcin, and procollagen I N-propeptide) and resorption (e.g., collagen type I crosslinked C-telopeptide—CTX [Beta-Crosslaps], serum C-telopeptides of type I collagen, and urinary N-telopeptides of type I collagen) [12].

An important factor significantly influencing changes in BMD and consequently, DXA result, is undoubtedly the hormonal regulation of the body. Several hormones affect bone mass, including glucocorticoids, estrogens, androgens, growth hormone (GH), parathyroid hormone (PTH), thyroid-stimulating hormone (TSH), and thyroxine (T4). Estrogen plays a pivotal role in maintaining BMD in women, with estrogen deficiency linked to increased bone resorption and a heightened risk of fractures [13]. Testosterone, GH, and insulin-like growth factor 1 (IGF-1) promote bone formation, whereas glucocorticoid excess simultaneously increases bone resorption and inhibits bone formation [14]. Androgen receptors, present in growth plate osteoblasts in both males and females, are thought to mediate the anabolic effects of testosterone on bone. However, estrogen is regarded as the more critical sex steroid for skeletal maturation and mineralization. The precise mechanism remains unclear, as it is not fully established whether estradiol acts directly on bone or indirectly through other mediators of bone growth [15].

Endocrinopathies may, therefore, represent an additional factor influencing bone mineral density and the attainment of peak bone mass. In female adolescents, a lower lumbar BMD has been observed in amenorrheic teens compared with their peers with regular menstruation. However, the difference is usually not significant when BMD is adjusted for body weight [15]. These findings suggest that nutritional status, including nutritionally dependent growth factors and gonadal status, may independently determine bone density.

During normal puberty, levels of GH and IGF-1 increase substantially, further amplified by rising sex steroid levels. Much of GH’s action on bone is mediated via IGF-1, which functions both endocrinologically and autocrine/paracrine as a bone trophic hormone. IGF-1 positively affects bone growth and turnover by stimulating osteoblasts, collagen synthesis, and longitudinal bone growth [15]. Among the various causes of low BMD and secondary osteoporosis, endocrine disorders—such as hypogonadism, cortisol excess, GH deficiency, hyperparathyroidism, and thyroid dysfunction (both hypo- and hyperthyroidism)—are major contributors [1,16].

Exploring relationships between BMD and pediatric endocrinopathies appears reasonable. In children with endocrine disorders, bone mass and potential mineralization disturbances may correlate with hormonal concentrations. Conversely, in children with conditions such as short stature or obesity, anthropometric parameters, and body composition may influence DXA results and potentially confound their clinical interpretation.

The aim of this study was to determine the actual prevalence of decreased BMD in children with various endocrinological disorders and to evaluate the impact of selected auxological and hormonal abnormalities on their BMD.

## 2. Materials and Methods

Approval for the study was obtained from the Bioethical Committee at the Polish Mother’s Memorial Hospital Research Institute (PMMH-RI) in Lodz, Poland.

The analysis included the medical records of 148 children with a mean age of 11.85 ± 3.34 years, comprising 73 girls (49.3%) and 75 boys (50.6%), who were hospitalized between 2020 and 2023 at the Department of Endocrinology and Metabolic Diseases at PMMH-RI in Lodz. Among the study participants, 34 children (22.9%) were diagnosed with obesity, 71 children (47.9%) with short stature, 15 children (10.1%) with precocious puberty, and 28 children (18.9%) with other diagnoses. These additional diagnoses included hypothyroidism (3 cases), hyperthyroidism (1 case), autoimmune thyroiditis (2 cases), menstrual irregularities (3 cases), hyperprolactinemia (2 cases), hyperandrogenism (2 cases), type 2 diabetes mellitus (1 case), reactive hypoglycemia (1 case), pituitary anomalies (5 cases), Cushing’s syndrome (1 case), gonadal dysgenesis (1 case), medium-chain acyl-CoA dehydrogenase (MCAD) deficiency (1 case), Addison disease (2 cases), and inflammatory bowel disease (3 cases).

Clinical data for each child included the primary diagnosis, height, body weight, and pubertal stage, as assessed by the Tanner scale [17]. Based on these parameters, the standard deviation score (SDS) for height (HSDS) was calculated. Additionally, body mass index (BMI) and BMI SDS values were derived using the appropriate percentile charts for the Polish pediatric population [18]. Given the significant proportion of children with short stature in the study group, the height age (HA) was determined for each child, defined as the age corresponding to the 50th percentile for their observed height

DXA was conducted using the Hologic Horizon Bone Densitometry System (MAN-04871-3402 version 007). For each patient, DXA assessments were performed at two sites: TBLH and the lumbar spine (Spine).

To minimize the impact of anthropometric variability on DXA results in the Spine projection, Bone Mineral Apparent Density (BMAD) was calculated for each patient using the formula: BMAD = BMC/BA^1.5 [19]. We employed an alternative normalization method for both projections to optimize the BMD Z-score. This method, which simultaneously accounts for both height and age, involves adjusting for the height-for-age Z-score (HAZ) [5,10]. Adjusted BMD Z-scores for age were calculated for both the Spine and TBLH projections.

The reference curves used for calculating age- and sex-specific BMD Z-scores were generated using the LMS (Lambda–Mu–Sigma) method, by Zemel et al. [10].

The adjustment method requires three steps: (1) a calculation of the aBMD for age Z-score and Height-Z-score (Ht-Z-score) (2) a calculation of a predicted aBMD Z-score based on age and Ht-Z-score; and (3) a calculation of the adjusted aBMD Z-score using the predicted aBMD Z-score and the aBMD for age Z-score.

Height-adjusted Z-scores were then calculated using the following equation:

BMD_HAZ_Z-score = BMDageZ-score − HAZ-predicted BMD Z-score

Height-Z-score, based on U.S. growth curves, can be calculated for example using the Epi Info Nutrition Calculator version 3.5.1, a free software package available for download from the U.S. Centers for Disease Control and Prevention web site (http://www.cdc.gov/epiinfo/, accessed on 20 June 2024) or (https://zscore.research.chop.edu/calcpedbonedens.php, accessed on 10 September 2024).

In all participants, serum concentrations of calcium, phosphate, alkaline phosphatase (ALP), 25-hydroxyvitamin D (25OHD), PTH, osteocalcin, and Crosslaps were measured. Additionally, in most cases, levels of TSH, fT4, fT3, IGF-1, IGF-binding protein 3 (IGF-BP3), morning cortisol, estradiol, testosterone, and bone age were assessed. IGF-1 levels were expressed as IGF-1 SDS, calculated using the Elmlinger reference standard [20], with reduced IGF-1 defined as IGF-1 SDS < −1.0.

All laboratory tests were conducted in the same PMMH-RI facility in Lodz using standardized methodologies. Serum osteocalcin and Crosslaps concentrations were measured using the electrochemiluminescence immunoassay (ECLIA) technique on a Roche Cobas automatic analyzer.

A statistical analysis of the collected data was performed using STATISTICA ver. 13.3 software (Statsoft, Cracow, Poland). The Shapiro–Wilk test was used to assess the normality of distribution, and the Levene’s test was used to assess equality of variance. Student’s t-test was applied for variables with a normal distribution, while non-parametric tests (Mann–Whitney U test or Kruskal–Wallis test) were used for variables with non-normal distributions. Pearson’s or Spearman’s correlation test was applied to evaluate relationships between variables. Descriptive statistics included the number of patients in specific groups as well as both the mean ± SD and median and interquartile ranges (median (Q1–Q3)) for continuous parameters were presented. Differences were considered statistically significant at *p* < 0.05.

## 3. Results

### 3.1. Prevalence of Bone Mineralization Disorders

**Figure 1:**
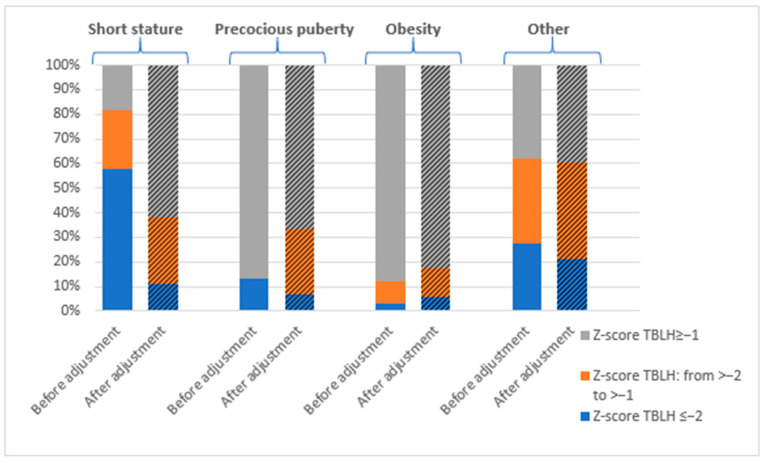
The prevalence of bone mineralization disorders expressed as a Z-score TBLH by diagnosis, before and after height adjustment (HAZ-adjusted Z-score; BMD_HAZ_ Z-score).

**Figure 2:**
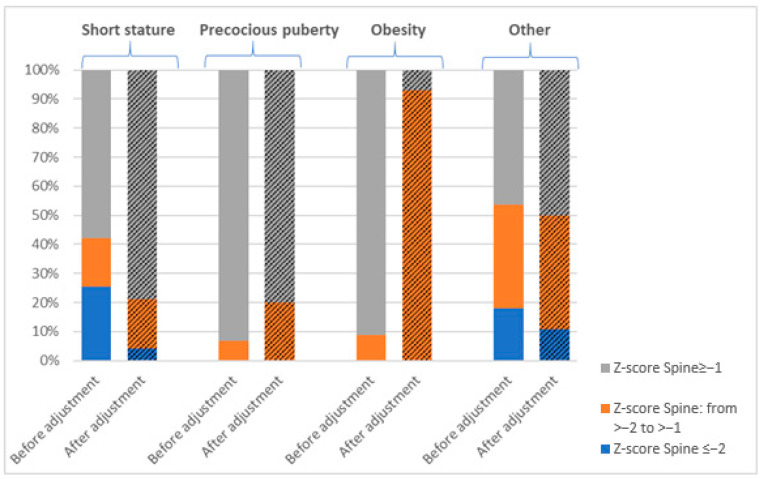
The prevalence of bone mineralization disorders expressed as a Z-score Spine by diagnosis, before and after height adjustment (HAZ-adjusted Z-score; BMD_HAZ_ Z-score).

A low bone mass (aBMD for age Z-score ≤ −2) was identified in 34.46% (51/148) of participants in the TBLH projection and 15.54% (23/148) in the Spine projection. Across the cohort, an aBMD for age Z-score < −1.0 was observed in 54.05% of children based on TBLH and 31.1% based on Spine measurements. The highest frequency of decreased BMD was noted in the short stature group, where 81.7% (58/71) exhibited aBMD for age Z-score < −1.0 in TBLH, and 42.3% (30/71) in Spine (Figure 1 and Figure 2).

After normalizing Z-scores for height (HAZ adjustment), the prevalence of low bone mass (aBMD_HAZ_ Z-score ≤ −2) decreased significantly to 11.4% (17/148) in the TBLH projection and 4.1% (6/148) in the Spine projection. Similarly, an aBMD_HAZ_ Z-score < −1.0 was identified in 37.2% (55/148) of participants in TBLH and 24.3% (36/148) in Spine.

In the short stature group, where initial assessments showed the highest frequency of bone mineralization disorders, the adjusting of Z-scores for height led to a marked reduction in abnormal findings. Specifically, the prevalence of an aBMD_HAZ_ Z-score < −1 decreased from 81.7% (58/71) to 38% (27/71) in TBLH (and from 42.3% (30/71) to 21.1% (15/71) in Spine (Figure 1 and Figure 2).

The lowest prevalence of bone mineralization disorders was observed in children with precocious puberty and obesity. None of the children in these groups were found to have a low bone mass in the Spine projection. In the TBLH projection, Z-scores ≤ −2 were identified in 13.33% of children with precocious puberty and 3.03% of those with obesity. After HAZ adjustment, a low bone mass was identified in 6.67% of children with precocious puberty and 5.88% of children with obesity in the TBLH projection (Figure 1 and Figure 2).

### 3.2. The Impact of Pubertal Stage on Bone Mineral Density in the Study Population

Pubertal signs were not observed in 54 children (36.5%) (Tanner stage I), early stages of puberty (stages II and III) were assessed in 44 children (29.7%), and advanced stages or the completion of puberty (stages IV and V) were identified in 50 children (33.8%). Therefore, pubertal characteristics were present in the majority of children (63.5%).

In the group of children with growth retardation, 43 children (60.6%) exhibited no signs of puberty, 26.76% were in stages II and III of puberty according to Tanner, and 12.68% were in stages IV and V of puberty. In children with precocious puberty, 12 children (80%) were in the early stages of puberty (stages II and III).

**Figure 3:**
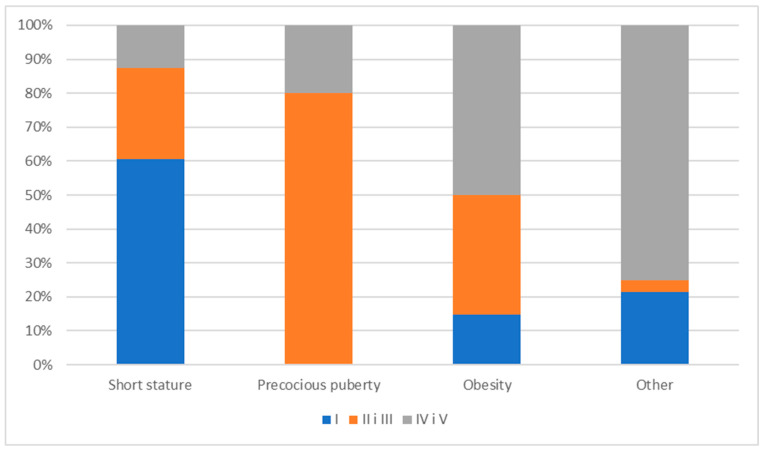
The distribution of pubertal stages according to the Tanner scale by diagnosis.

Among the children with obesity, 5 children (14.7%) were in the prepubertal stage, 12 children (35.3%) had puberty assessed as stages II or III according to the Tanner scale, and 17 children (50%) were in advanced puberty (stages IV and V) (Figure 3).

Children in the prepubertal stage had a significantly lower BMC Spine, BMC TBLH, aBMD Spine, aBMD TBLH, and BMAD Spine compared to those who had already entered puberty (*p* < 0.05). However, in the entire study group, BMD_HAZ_ Z-scores for TBLH and Spine did not differ significantly between the prepubertal group (Tanner stage I, 54/148) and those who had started puberty (Tanner stages II–V, 94/148) (for Z-score _HAZ_ TBLH *p* = 0.55, for Z-score _HAZ_ Spine *p* = 0.77).

In the group of children with growth retardation, 9.9% (7/71) were diagnosed with growth hormone deficiency (somatotropin deficiency), while the rest exhibited normal growth hormone secretion. Reduced IGF-1 (IGF-1 SDS < −1) was observed in 46.5% (33/71) of the children with growth retardation. Among children with obesity, no cases of hypercortisolism were found.

In the group of children with precocious puberty, 6 of 15 patients were diagnosed with central precocious puberty, while the remaining cases were mild variants that required observation but no treatment.

### 3.3. A Comparison of Anthropometric Parameters, Laboratory Indicators of Calcium–Phosphate and Hormonal Metabolism, and DXA Parameters by Patient Diagnosis

The analyzed patient groups differed significantly in terms of age, height expressed as HSDS, and BMI SDS (Table 1). The youngest group of patients consisted of children with precocious puberty and growth retardation, while the oldest children were in the group with other diagnoses.

Serum concentrations of PTH, 25OHD, osteocalcin, Crosslaps, TSH, fT3, fT4, cortisol, and estradiol did not differ significantly between the groups (*p* > 0.05) (Table 2). However, it should be emphasized that each of the groups contained a few individual cases of thyroid dysfunction, adrenal gland disorders, and hypogonadism, which could have affected the results of our study.

Children with growth retardation showed significantly lower levels of IGF-1 and, among the DXA parameters, had a lower BMC Spine, aBMD Spine, BMAD, and BMD TBLH compared to children with obesity. For aBMD for age Z-score Spine and aBMD for age Z-score TBLH, this difference was also statistically significant among the children with growth retardation and those with obesity and precocious puberty. After adjusting the Z-score for height, no significant differences were observed in either the Spine or TBLH projections between children with growth retardation and the other groups (Table 3).

### 3.4. An Analysis of Factors Influencing Bone Mineral Density and DXA Parameters in the Entire Study Group of Patients

A significant positive correlation was found between almost all DXA parameters (BMC Spine, aBMD Spine, BMAD, BMC TBLH, and aBMD TBLH) and the following factors: calendar age, HSDS, BMI SDS, estradiol, IGF-1, and IGF BP3. Additionally, serum testosterone levels positively correlated with the above-mentioned DXA parameters, except for BMAD in both girls and boys (Table 4).

IGF-1 SDS was positively correlated with the following DXA parameters: aBMD for age Z-score Spine (r = +0.43), aBMD _HAZ_ Z-score Spine (r = +0.28), aBMD for age Z-score TBLH (r = +0.49), and aBMD _HAZ_ Z-score TBLH (r = +0.43). These correlations are detailed in Table 4.

Vitamin D deficiency, defined as a 25OHD level < 30 ng/mL, was observed in 104 children (69.8%). Of these, 44 children had levels < 20 ng/mL, and 60 had levels between 20–30 ng/mL. Surprisingly, there was a negative correlation between vitamin D levels and all DXA parameters (BMC Spine, BMD Spine, BMAD, aBMD for age Z-score Spine, aBMD_HAZ_Z-score Spine, BMC TBLH, aBMD TBLH, aBMD for age Z-score TBLH, and aBMD _HAZ_ Z-score TBLH).

Adequate levels of 25OHD in serum (30–50 ng/mL) were found in the following: twenty children with growth retardation (29.4%), three children with precocious puberty (20%), four children with obesity (12.5%), and six children with other diagnoses (24%). However, no statistically significant differences in the average vitamin D levels were found between the groups (Table 1).

Serum calcium and phosphate levels, as well as the calcium-to-creatinine ratio from morning urine samples, had a significantly negative impact on bone mass. Additionally, serum markers of bone turnover (osteocalcin and Crosslaps) negatively influenced bone mass.

No statistically significant correlations were found between serum levels of PTH, TSH, fT4, or cortisol and any DXA parameters in the patient groups. There was a significant negative correlation between serum fT3 levels and BMD Spine and BMAD, suggesting that fT3 levels might influence bone mineral density in this cohort.

### 3.5. An Analysis of Factors Affecting Bone Mineral Density in Children with Growth Retardation

**Figure 4:**
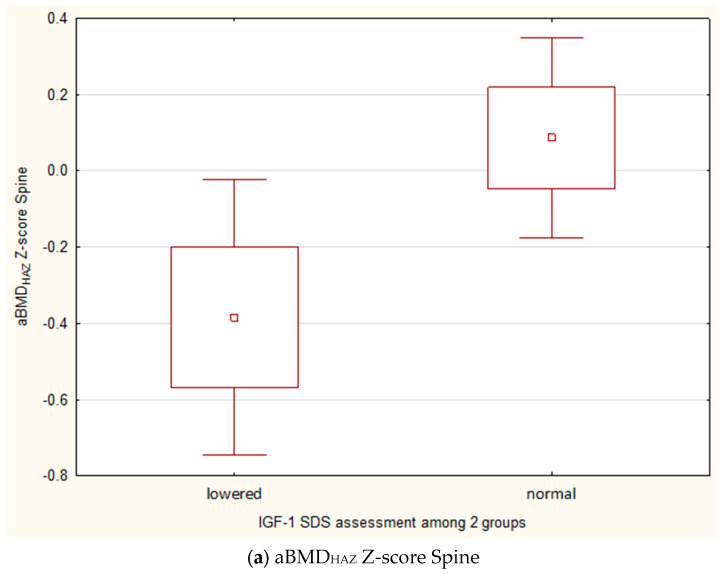

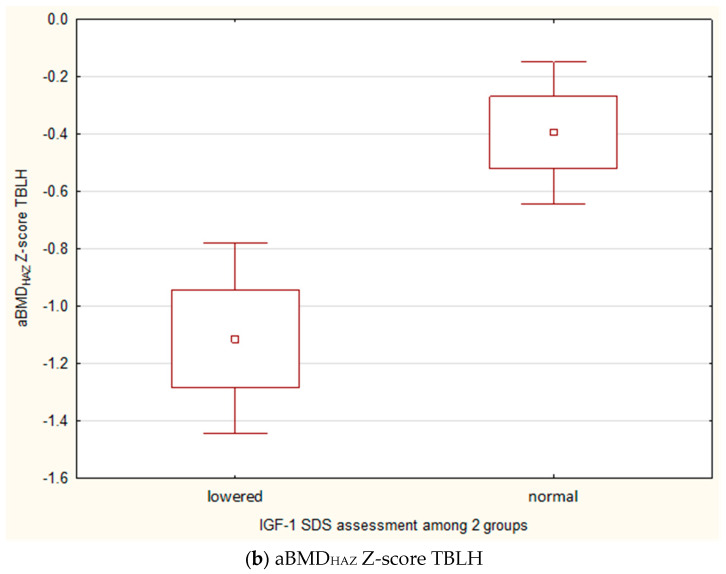
A comparison of aBMD_HAZ_ Z-score Spine (**a**) and TBLH (**b**) in growth retardation patients according to IGF-1 SDS.

In the group of children with growth retardation (N = 71), which constituted the largest group of patients, a statistically significant lower value of both aBMD_HAZ_ Z-score Spine and aBMD_HAZ_ Z-score TBLH was observed in patients with decreased IGF-1 SDS (IGF-1 SDS < −1) compared to those with normal IGF-1 SDS (Figure 4a,b).

**Figure 5:**
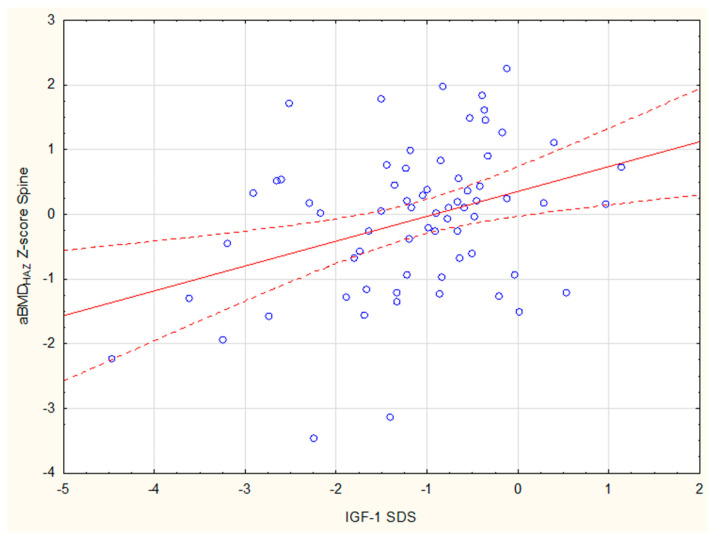
The correlation between IGF-1 SDS and aBMD_HAZ_ Z-score Spine among children with growth retardation.

**Figure 6:**
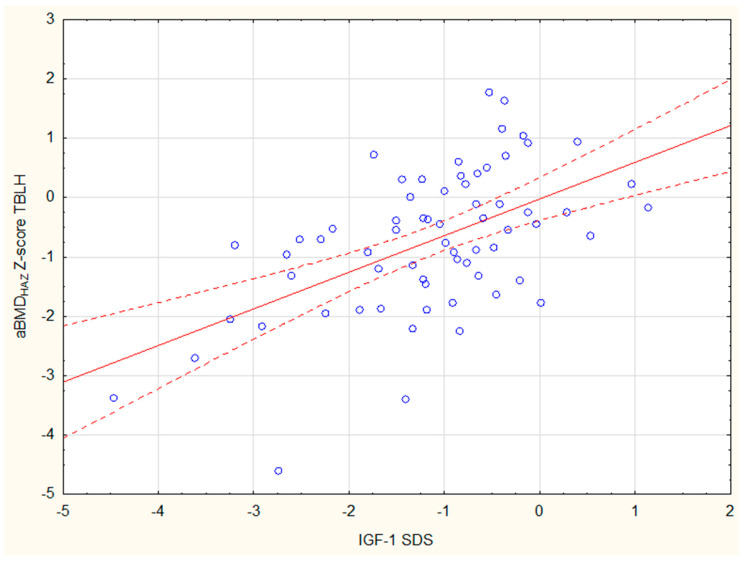
The correlation between IGF-1 SDS and aBMD_HAZ_ Z-score TBLH among children with growth retardation.

Among children with growth retardation, a statistically significant positive correlation was found between the adjusted Z-score Spine and both BMI SDS (r = 0.4) and IGF-1 SDS (r = 0.35) (Figure 5). A similar relationship existed between the aBMD_HAZ_ Z-score TBLH and both BMI SDS (r = 0.37) and IGF-1 SDS (r = 0.55) (Figure 6).

In this group of children with growth retardation, no significant negative correlation was observed between 25OHD levels, bone turnover markers (Crosslaps and osteocalcin), and the adjusted Z-scores for Spine and TBLH, which was seen in the entire cohort of the study.

## 4. Discussion

The formation of peak bone mass is influenced by numerous genetic, environmental, and hormonal factors. Considering the fact that the growth period is particularly dynamic in terms of both growth processes and hormonal changes, it is natural to consider children with endocrine disorders as a high-risk group for bone mineralization disorders. Identifying factors that negatively impact these processes, leading to abnormally low bone mass, is critical.

Hormonal deficiencies, such as growth hormone deficiency, hypercortisolism due to Cushing’s syndrome (both ACTH-dependent and ACTH-independent), thyroid disorders (hyperthyroidism and hypothyroidism), hyperparathyroidism, and hypogonadism, are some of the most commonly mentioned endocrine disturbances affecting bone mineral density.

In our study group, DXA results showed low bone mass (aBMD Z-score ≤ −2) in 34.46% of patients (51/148) in the TBLH projection and 15.54% of children (23/148) in the Spine projection. Additionally, aBMD for age Z-scores < −1 were found in 54.05% of children in the TBLH projection and 31.1% in the Spine projection.

It is noteworthy that the group of children with growth retardation had the highest incidence of bone mineralization disorders. In the TBLH projection, aBMD for age Z-scores < −2 was found in 57.75% of cases, and aBMD for age Z-scores between −1 and −2 was observed in 23.94%. In the Spine projection, aBMD for age Z-scores < −2 was found in 25.35%, and aBMD for age Z-scores between −1 and −2 was found in 16.90%.

Numerous publications suggest that growth hormone deficiency may contribute to disturbances in bone mineralization [15,21].

After adjusting the DXA results for height in the entire studied group, it was found that the incidence of low bone mass was significantly lower. Following the normalization of the results to height age, low bone mass was observed in only 11.2% of children with short stature in the TBLH projection and 4.23% in the Spine projection. The height-adjusted Z-scores in both projections in children with short stature did not differ significantly from the other patient groups. Thus, in accordance with the findings of other studies, it can be concluded that height and body mass largely influence DXA results, and it is particularly necessary to normalize results for height in patients with growth disorders.

Numerous studies published to date have indicated a positive correlation between BMI and BMD [22,23], suggesting that children diagnosed with obesity exhibit better bone mineral density compared to children with normal body weight. Our study also found a statistically significant positive correlation between height, expressed as HSDS, and BMI SDS with the following parameters: BMC Spine, BMD Spine, BMAD, BMC TBLH, and BMD TBLH.

Adjusting DXA results according to anthropometric measurements is particularly important in children with short stature. Högler and Shawn emphasized in their study that relying solely on Z-score BMD adjustments for age and gender, without considering anthropometric measurements (including bone size), has led to the misconception that children with growth hormone deficiency (GHD) exhibit a lower bone mineral density and an increased risk of fractures [24]. Similarly, our study demonstrated that after normalizing the Z-score BMD for height, the incidence of bone mineralization disorders in children with short stature was not significantly different from that observed in other groups. Högler suggests that “using appropriate size-corrections, bone density is normal in children and adults with isolated GHD” [24]. Cortical density, trabecular density, and trabecular volume are normal when measured by peripheral quantitative computerized tomography and histomorphometry. The only verifiable deficit affects cortical thickness (periosteal expansion), which the authors link to the mechanostat theory. This suggests that the GHD-induced deficit in muscle force secondarily causes low cortical thickness. There is no evidence that isolated childhood-onset GHD [24,25], or severe GH resistance, increases fracture risk in children. The authors also note that “density is just one of several variables that affect structural bone strength” [24].

On the other hand, the GH/IGF-1 axis is essential for normal longitudinal bone growth and bone mass acquisition in association with sex steroids. GH not only directly promotes the differentiation of mesenchymal stem cells (MSC) into osteoblasts but also stimulates osteoblast proliferation [26]. GH may indirectly promote osteoblast differentiation by upregulating bone morphogenetic proteins (BMPs) and IGF-1/IGF-2B [27]. IGF-1, primarily produced in the liver, exerts stimulatory effects by binding to IGF-1 receptors on osteoblasts, resulting in cell differentiation [28], and its serum levels are positively correlated with BMD in older women and men [29]. Therefore, we sought to determine whether IGF-1 levels have any impact on the bone mass in children with short stature or whether bone mass is primarily dependent on the child’s height.

Numerous reports indicate a positive correlation between IGF-1 levels and bone mass in healthy children [30,31,32]. Similarly, in our study, IGF-1 and IGF-BP3 levels were significantly positively correlated with nearly all parameters of bone mineral density (BMC Spine, BMD Spine, BMAD Spine, BMC TBLH, and BMD TBLH). IGF-1 SDS was also positively correlated with aBMD Z-score Spine and aBMD Z-score TBLH both before and after adjusting to height. An additional indicator confirming the role of growth hormone and IGF-1 in peak bone mass development is the fact that in the group of children with short stature, those with decreased IGF-1 SDS levels exhibited significantly lower aBMD Z-scores (both for Spine and TBLH) compared to children with short stature whose IGF-1 SDS levels were within the normal range.

Kim et al., studying children diagnosed with leukemia, showed that IGF-1 SDS was significantly lower in children with low BMD compared to those without low BMD (−1.23 ± 0.817 vs. −0.72 ± 0.779, *p* = 0.001). Moreover, no significant differences were found between the two groups in terms of age at leukemia diagnosis, transplantation, total body irradiation (TBI), calcium levels, and 25-hydroxyvitamin D levels [33]. These authors suggest that IGF-1 could be an important indicator of bone mineralization disorders in children after the completion of leukemia treatment [33].

Body height plays a critical role in the accurate interpretation of DXA measurements. Therefore, in our study, height-adjusted DXA Z-scores were calculated for all participants, irrespective of their underlying condition. Among the studied population, 10 children presented with a height standard deviation score (HSDS) exceeding +2. None of these individuals showed clinical or biochemical signs of growth hormone excess, and thus gigantism was not diagnosed in any case. Furthermore, none of the tall patients demonstrated low bone mass, either prior to or following height adjustment of DXA results

The lowest incidence of low bone mass in our study was observed among children with obesity and precocious puberty. Among the children with obesity, none were diagnosed with low bone mass based on Z-score BMD Spine, and in the TBLH projection, BMD_HAZ_Z-score < −2 was found in only 5.88% of the children. Growing evidence suggests that adiposity influences bone health in children. Previous studies have shown that children and adolescents with obesity have a higher BMC than normal-weight peers, indicating that adipose tissue exerts a positive effect on bone structure [34,35]. Additionally, body adiposity represents a mechanical load that is beneficial for bone accrual [36]. However, several studies have shown a reduction in bone mass in obese children, although there is still debate on this issue. It has been reported that children with obesity have an increased rate of extremity fractures, suggesting poorer bone quality [37,38]. Both adipocytes and osteoblasts derive from multipotent mesenchymal stem cells (MSCs), and obesity drives the differentiation of MSCs toward adipocytes at the expense of osteoblast differentiation. Furthermore, adipocytes in the bone marrow microenvironment release pro-inflammatory and immunomodulatory molecules that upregulate osteoclast formation and activation, thus promoting bone fragility.

In our studied group, children with obesity had a significantly higher BMC Spine, aBMD Spine, BMAD, aBMD TBLH, aBMD for age Z-score Spine, and aBMD for age Z-score TBLH compared to children with short stature (but after adjustment for height, these associations became statistically insignificant). Therefore, our findings could contribute further to the ongoing discussion about the impact of obesity on bone mineral density. Correcting DXA results for anthropometric measurements seems essential to avoid skewing the results. However, our study group is small, and thus we cannot draw far-reaching conclusions. BMC is recommended as the best parameter to assess bone mass status in children and adolescents, but neither the use of DXA nor the research conducted has led to a consensus on the topic [38].

BMD increases significantly during puberty; it is well established that approximately 40% of peak bone mass is acquired between Tanner stages II and V [39,40], with the rate of acquisition being particularly high between stages III and IV [41,42]. Subsequently, bone acquisition slows down, and the increase continues at a slow rate until bone consolidation is completed. Gonadal steroids can influence bone mass acquisition either directly or indirectly through their effects on molecules such as growth hormone, insulin-like growth factor-1, and 1,25(OH)2D [27]. In our study, children in the prepubertal phase had a significantly lower BMC Spine, BMC TBLH, BMD Spine, BMD TBLH, and BMAD Spine compared to children in puberty.

On the other hand, aBMD Z-score TBLH and aBMD Z-score Spine did not show any significant statistical differences before or after adjustment between the prepubertal group (stage I according to Tanner) and those who had started puberty (stages II–V according to Tanner). The effect of estrogen and androgen on peak bone mass development is well established, but it is uncertain whether precocious puberty in children significantly affects bone mineral density parameters, especially considering the frequent acceleration of growth and bone age in this group, which are variables that can influence the interpretation of DXA results.

Children with precocious puberty, along with those with obesity, represented another group in which bone mineralization disorders were least commonly observed. After adjusting aBMD Z-scores to height, none of the children with precocious puberty had low bone mass in the Spine projection, and only 6.67% of children showed low bone mass in the TBLH projection.

In a study by Alessandri et al., which examined girls with central precocious puberty, lumbar spine bone mineral density was slightly higher in individuals diagnosed with precocious puberty compared to controls. However, this tendency disappeared after correction for bone age [42]. In our study, DXA parameters (BMC Spine, aBMD Spine, BMAD, BMC TBLH, and aBMD TBLH) did not differ significantly between children with precocious puberty and those in the other study groups, including children with short stature, obesity, and other diagnoses. However, it should be noted that our cohort of children with precocious puberty included those with central precocious puberty as well as those with milder forms of early puberty. Furthermore, we adjusted the results based only on the patients’ height, not their bone age. However, bone age did not differ significantly between groups, and in the entire cohort, a positive correlation was found between bone age and BMC Spine, BMD Spine, BMAD, and BMC TBLH.

Brazilian researchers have demonstrated that GnRH agonist treatment seems to have no detrimental effect on bone mineral density [42]. On the other hand, it is widely recognized that estrogens play a crucial role in bone metabolism, stimulating bone formation and reducing its resorption [43,44]. In our study, a statistically significant positive correlation was found between estradiol levels and BMC Spine, BMD Spine, BMAD, BMC TBLH, and BMD TBLH. A similar relationship was also observed between testosterone levels and BMC Spine, BMD Spine, BMC TBLH, and BMD TBLH. There is a close relationship between adipose tissue and estrogen metabolism. Indeed, adipose tissue is one of the major sources of aromatase, which converts testosterone into estrogens. Higher serum concentrations of estrogens have been found in obese postmenopausal women compared to their non-obese counterparts [45].

Gonadal function plays a crucial role in the development of peak bone mass in children. In our study cohort, a notable number of patients presented with precocious puberty; however, only a single case of delayed puberty was observed—a girl diagnosed with hypogonadotropic hypogonadism in the context of panhypopituitarism. This patient demonstrated a significantly reduced bone mass: Spine Z-score: −4.0; Spine HAZ Z-score: −3.46; TBLH Z-score: −2.7; TBLH HAZ Z-score: −1.95. These findings suggest that patients with hypogonadism represent a distinct subgroup that requires careful DXA assessment. We intend to address this in future research with a dedicated focus on this population.

In conclusion, among the factors positively influencing the DXA parameters in our patients, body height expressed as HSDS, BMI SDS, IGF-1 levels, estradiol, and testosterone levels in serum were identified, regardless of the patients’ gender.

When analyzing the relationship between individual hormones and bone mass in our study, we also considered the potential impact of TSH levels and free thyroid hormones on DXA parameters. The influence of abnormal thyroid hormone and TSH levels, whether under subclinical or overt conditions, on low BMD in children and adolescents, remains controversial. However, it should be emphasized that the vast majority of the children in our analysis were euthyroid.

In our study, a negative correlation was found between bone turnover markers—osteocalcin (a marker of bone formation) and Crosslaps (a marker of bone resorption)—and nearly all bone mineral density parameters (BMC Spine, BMD Spine, BMAD, BMC TBLH, and BMD TBLH). Bone biomarkers are specific markers that reflect both bone formation by osteoblasts and resorption by osteoclasts. Children have significantly higher concentrations of bone biomarkers than adults due to both skeletal growth and the rapid bone turnover that occurs during childhood and adolescence [8]. Previous pediatric studies have demonstrated that serum osteocalcin is a sensitive marker of skeletal growth in healthy children and those with pathologically increased growth velocity [46]. Osteocalcin, the major non-collagenous protein synthesized by osteoblasts, plays an important role in regulating bone growth and the correct deposition of minerals in the bone matrix. Its expression follows the proliferative phase of osteoblastic differentiation, so it can be considered a marker of mature osteoblasts. Serum levels of osteocalcin and deoxypyridinoline are not stable throughout life and are higher in infants and children than in adults. Peak values occur during puberty [47]. Paldanius et al., in their study of over 170 children, noted that all osteocalcin values, along with other bone turnover markers, increased with puberty and correlated with pubertal growth, which occurred earlier and declined sooner in girls than in boys. The authors of this study emphasize that in children and adolescents, circulating and urinary osteocalcin reflect growth status more accurately than bone mineral density (BMD) or body composition [48]. Similarly, in the study by El-Dorry et al., osteocalcin was found to be negatively correlated with most DXA results (area, BMC, BMD, lean mass, and Z-score) in obese children compared to their non-obese peers [47].

β-CTX is released into the bloodstream during bone resorption by osteoclasts through the degradation of type I collagen. It is a sensitive marker that reflects osteoclast activity and is internationally recognized as a marker for bone resorption [49]. Different trends in β-CTX levels have been observed between girls and boys. In boys, from preschool through adolescence, β-CTX levels increased with advancing age, and a positive correlation between age and β-CTX was found. In girls, β-CTX levels increased from preschool to preadolescence but did not change significantly from preadolescence to adolescence, reaching a plateau. The sex differences in bone development are closely related to the development of puberty and the impact on bone size. Due to earlier skeletal maturation, the degree of bone resorption in girls reached a plateau compared to boys [49]. Korpysz et al., in a study of children with SGA treated with growth hormone during the prepubertal period, found a statistically significant positive correlation between the child’s height after 6 and 12 months of GH therapy and CTX levels, highlighting that this marker could also serve as a predictor of GH treatment outcomes [50]. This confirms previous observations that bone turnover markers in the pubertal age group better reflect the remodeling process associated with growth rather than fracture risk, as seen in the adult population. Furman et al., similar to our study, found a negative correlation between lumbar BMD and CTX in patients with Addison’s disease [51].

However, our results yielded surprising findings when analyzing the effect of vitamin D, which is widely used in the prevention and treatment of bone mineralization disorders. Our study revealed a statistically significant negative correlation between serum 25OHD levels and all DXA parameters (BMC Spine, BMD Spine, BMAD, BMC TBLH, BMD TBLH, aBMD Z-score Spine, and TBLH, both before and after adjustment for height). There are reports suggesting a beneficial effect of vitamin D on bone health in children [52] and adults [53,54]. However, other studies have not found any associations between 25OHD levels and BMD or fracture risk [55,56]. A 2023 analysis in Lancet Diabetes and Endocrinology highlighted that the concurrent use of vitamin D and calcium supplements does not reduce the risk of any type of fracture compared to patients receiving placebo or no treatment. The authors of this study emphasize that recent research points to new, unexpected side effects of vitamin D supplementation (beyond the well-known hypercalcemia, hypercalciuria, and nephrocalcinosis), including increased rates of fractures, falls, and hospitalizations [57]. Therefore, our findings, indicating a negative correlation between vitamin D levels and bone mineral density parameters, provide an interesting contribution to the ongoing discussion.

## 5. Conclusions

In summary, our findings emphasize that patients with endocrine disorders are at risk for developing bone mineralization disorders during childhood. We acknowledge that the “others” diagnosis group is heterogeneous; however, we believe that the analysis results remain valuable and provide meaningful insights. Low bone mass was most frequently observed in patients with short stature and least often in those with obesity and precocious puberty. It should be noted that patient height significantly influences DXA results, so in patients with short stature or tall stature, the adjusting of DXA parameters to the patient’s height is necessary. Failure to normalize could lead to an overdiagnosis of bone mineralization disorders in children with short stature and potentially unnecessary therapy. However, factors positively influencing bone mineral density include patient height expressed as HSDS, BMI-SDS, IGF-1, estradiol, and testosterone levels, while factors negatively affecting peak bone mass include bone turnover markers (CTX and osteocalcin) and serum 25OHD levels.

The uniqueness of our study lies in its clear demonstration of how critically important height adjustment is in the interpretation of DXA results in the pediatric population. This aspect is particularly significant in children with short stature, as a failure to correct for height can lead to overdiagnosis of bone mineralization disorders and, consequently, to unnecessary therapeutic interventions.

Although we recognize that the need to normalize DXA results for height is well established and incorporated into the official ISCD guidelines, this approach is still not consistently applied in everyday clinical practice. We hope our work will contribute to raising awareness and improving adherence to this important standard among clinicians.

## Figures and Tables

**Table 1:** A comparison of anthropometric parameters and laboratory indicators assessing calcium–phosphate metabolism by patient diagnosis (*#^—*p* < 0.05).

Diagnosis	Short Stature Mean ± SD Median (Q1–Q3)	Precocious Puberty Mean ± SD Median (Q1–Q3)	Obesity Mean ± SD Median (Q1–Q3)	Other Mean ± SD Median (Q1–Q3)
Age [years]	10.96 ± 3.07 * 11.16 (8.59–13.31)	9.58 ± 1.68 # 9.50 (8.58–10.70)	12.74 ± 3.12 # 13.34 (11.30–14.60)	14.22 ± 3.35 *# 15.14 (12.76–16.15)
HSDS	−2.56 ± 1.16 *#^ −2.56 (−3.13–(−2.0))	0.14 ± 1.26 * 0.08 (−0.92–1.05)	0.74 ± 1.47 # 0.94 (-0.29–1.67)	−0.45 ± 1.75 ^ −0.17 (−1.57–0.52)
BMI SDS	−0.42 ± 2.51 * −0.75 (−1.91–0.12)	0.57 ± 2.26 # 0.16 (−1.24–0.68)	6.22 ± 4.12 *#^ 6.37 (3.42–8.58)	−0.51 ± 1.56 ^ −0.73 (−1.85–0.68)
Serum calcium [mmol/L]	2.41 ± 0.10 * 2.41 (2.34–2.47)	2.42 ± 0.13 2.41 (2.34–2.50)	2.40 ± 0.11 2.41 (2.31–2.47)	2.33 ± 0.14 * 2.32 (2.23–2.42)
Serum phosphate [mmol/L]	1.75 ± 0.18 1.74 (1.63–1.88)	1.79 ± 0.16 1.81 (1.64–1.86)	1.69 ± 0.27 1.72 (1.51–1.90)	1.61 ± 0.23 1.57 (1.49–1.76)
Serum Magnesium [mmol/L]	0.84 ± 0.06 0.84 (0.81–0.87)	0.83 ± 0.05 0.83 (0.79–0.86)	0.84 ± 0.07 0.84 (0.78–0.86)	0.81 ± 0.07 0.80 (0.76–0.84)
ALP [U/L]	153.33 ± 33.72 152.00 (119.00–179.00)	205.17 ± 60.39 * 194.00 (163.00–270.00)	205.71 ± 92.01 # 217.00 (98.00–302.00)	88.60 ± 29.07 *# 78.00 (75.00–96.00)
Ca/Cr ratio	0.11 ± 0.08 0.09 (0.03–0.16)	0.12 ± 0.12 0.08 (0.03–0.15)	0.08 ± 0.10 0.05 (0.03–0.10)	0.10 ± 0.07 0.10 (0.05–0.15)
PTH [pg/mL]	38.73 ± 104.28 25.15 (19.90–30.50)	32.69 ± 10.33 32.30 (26.60–40.70)	32.26 ± 8.95 32.00 (24.90–35.80)	37.75 ± 30.35 30.25 (25.70–38.40)
25OHD [ng/mL]	28.43 ± 13.37 26.05 (20.40–32.60)	21.08 ± 11.05 17.50 (11.50–29.70)	21.45 ± 7.03 20.40 (17.25–25.97)	25.98 ± 10.25 23.70 (19.60–28.10)
Osteocalcin [ng/mL]	81.68 ± 27.98 75.10 (62.00–97.20)	109.03 ± 32.38 97.10 (83.70–142.40)	76.34 ± 35.10 79.55 (39.80–100.50)	80.46 ± 59.16 64.90 (41.10–99.40)
Crosslaps [pg/mL]	1889.69 ± 506.57 1939.00 (1571.00–2185.00)	2176.67 ± 502.03 2136.00 (1951.00–2532.00)	1679.57 ± 694.04 1580.50 (1027.0–2283.00)	1719.15 ± 843.80 1654.00 (1044.0–2096.00)

*, #, ^—values marked with the same symbols in the same row differ significantly from each other in terms of the given *p* value.

**Table 2:** A comparison of laboratory indicators assessing hormonal metabolism by patient diagnosis (*#—*p* < 0.05).

Diagnosis	Short Stature Mean ± SD Median (Q1–Q3)	Precocious Puberty Mean ± SD Median (Q1–Q3)	Obesity Mean ± SD Median (Q1–Q3)	Other Mean ± SD Median (Q1–Q3)
TSH [uIU/mL]	2.51 ± 1.02 2.45 (1.72–3.07)	2.73 ± 1.35 2.55 (1.66–3.52)	2.42 ± 1.26 2.06 (1.59–3.33)	2.27 ± 1.66 1.94 (1.28–3.18)
fT4 [ng/dL]	1.24 ± 0.17 1.22 (1.14–1.35)	1.27 ± 0.20 1.30 (1.12–1.41)	1.16±0.16 1.11 (1.04–1.26)	1.27 ± 0.35 1.22 (1.11–1.32)
fT3 [pg/mL]	3.85 ± 0.60 3.76 (3.44–4.11)	4.11 ± 0.67 4.12 (3.63–4.22)	3.82 ± 0.60 3.79 (3.42–4.10)	3.57 ± 0.78 3.43 (3.07–4.03)
Estradiol	18.88 ± 28.36 5.00 (5.00–20.40)	18.27 ± 18.20 7.90 (5.00–32.80)	29.37 ± 32.97 19.85 (5.00–42.40)	39.88 ± 47.35 25.10 (7.10–42.90)
F: N = 55	N = 17	N = 10	N = 17	N = 11
M: N = 31	F: 25.16 ± 34.20 5.00 (5.00–43.30)	F: 19.18 ± 19.64 8.30 (5.00–38.10)	F: 38.68 ± 37.46 41.60 (14.40–44.20)	F: 55.05 ± 57.57 28.90 (6.10–94.20)
[pg/mL]	N = 11 M: 9.15 ± 11.3 5.00 (5.00–7.00)	N = 3 M: 15.23 ± 15.28 7.90 (5.00–32.80)	N = 9 M:11.79 ± 7.57 7.90 (5.00–18.40)	N = 8 M: 19.03 ± 13.03 15.25 (9.05–27.85)
Testosterone				
F: N = 46	0.84 ± 1.42 0.075 (0.025–1.35)	0.85 ± 2.00 0.06 (0.03–0.14)	0.97 ± 1.23 0.48 (1.20–1.23)	1.93 ± 2.54 0.31 (0.12–4.23)
M: N = 51	F: 0.17 ± 0.15 0.12 (0.04–0.26)	F: 0.053 ± 0.04 * 0.04 (0.03–0.07)	F: 0.04 ± 0.05 * 0.30 (0.11–0.63)	F: 0.22 ± 0.18 0.14 (0.10–0.27)
[ng/mL]	N = 10	N = 10	N = 16	N = 10
	M: 1.11 ± 1.61 * 0.08 (0.03–1.74) N = 25	M: 2.84 ± 3.15 2.25 (0.15–5.52) N = 4	M: 1.72 ± 1.55 1.13 (0.45–3.06) N = 12	M: 3.65 ± 2.65 * 4.23 (1.17–5.71) N = 10
IGF-1 SDS	−1.13 ± 1.05 * −0.95 (−1.66–(−0.46))	0.10 ± 1.02 * −0.08 (−0.63–0.54)	−0.52 ± 1.08 −0.41 (−1.49–0.24)	−0.90 ± 1.05 −0.71 (−1.40–(−0.31))
IGF-1 [ng/mL]	179.01 ± 88.30 *# 161.25 (110.90–221.40)	313.00 ± 175.69 325.80 (168.40–418.60)	285.44 ± 109.17 * 246.90 (190.40–380.60)	276.56 ± 122.72 # 284.65 (173.40–362.60)
IGF-BP3 [ng/mL]	3887.50 ± 924.49 3774.50 (3250.00–4429.00)	4211.67 ± 719.06 4496.00 (3564.00–4749.00)	4489.75 ± 1009.62 4693.50 (4178.0–4883.00)	4156.21 ± 861.12 4139.00 (3089.0–4688.00)
Bone age [years]	9.44 ± 3.48 10.00 (6.00–12.25)	9.96 ± 2.15 10.00 (8.20–11.00)	12.72 ± 2.88 13.00 (11.00–14.50)	12.38 ± 4.35 14.00 (12.00–15.00)
Cortisol [ug/dL]	13.81 ± 5.07 14.40 (10.70–17.10)	13.72 ± 3.03 14.00 (12.80–15.50)	13.53 ± 4.37 13.40 (10.80–16.70)	11.62 ± 4.42 11.20 (7.50–14.70)

*, #—values marked with the same symbols in the same row differ significantly from each other in terms of the given *p* value.

**Table 3:** A comparison of DXA parameters by patient diagnosis (*#^—*p* < 0.05).

Diagnosis	Short Stature Mean ± SD Median (Q1–Q3)	Precocious Puberty Mean ± SD Median (Q1–Q3)	Obesity Mean ± SD Median (Q1–Q3)	Other Mean ± SD Median (Q1–Q3)
BMD Spine [g/cm^2^]	0.58 ± 0.14 * 0.55 (0.49–0.64)	0.63 ± 0.11 0.61 (0.54–0.71)	0.85 ± 0.21 *# 0.86 (0.65–1.03)	0.79 ± 0.21 # 0.81 (0.65–0.95)
aBMD for age Z-score Spine	−1.19 ± 1.19 *# −1.00 (−2.00–(−0.40))	0.28 ± 1.06 * 0.30 (−0.50–0.70)	0.80 ± 1.16 # 0.95 (0.20–1.30)	−0.79 ± 1.66 −1.00 (−1.70–0.50)
aBMD_HAZ_ Z-score Spine	−0.087 ± 1.13 0.10 (−0.93–0.54)	0.08 ± 1.03 0.01 (−0.77–0.64)	0.36 ± 1.04 * 0.30 (−0.27–0.94)	−0.77±1.46 * −0.94 (−1.38–0.39)
BMAD	0.10 ± 0.02 *# 0.09 (0.08–0.11)	0.10 ± 0.01 0.10 (0.08–0.11)	0.12 ± 0.02 * 0.12 (0.10–0.14)	0.11 ± 0.02 # 0.11 (0.09–0.13)
BMC Spine [g]	21.99 ± 9.21 *# 20.42 (16.23–24.64)	24.84 ± 6.52 22.78 (20.62–31.77)	45.18 ± 17.94 * 44.97 (29.35–57.00)	42.16 ± 17.85 # 43.13 (27.63–55.67)
BMD TBLH [g/cm^2^]	0.63 ± 0.12 *# 0.62 (0.55–0.69)	0.67 ± 0.09 0.65 (0.60–0.73)	0.86 ± 0.14 * 0.86 (0.75–0.96)	0.81 ± 0.15 # 0.87 (0.71–0.90)
aBMD for age Z-score TBLH	−2.19 ± 1.39 *#^ −2.20 (−2.90–(−1.20))	−0.29 ± 1.11 * −0.40 (−0.90–0.20)	0.41 ± 1.15 # 0.40 (−0.10–1.20)	−1.24 ± 1.39 ^ −1.10 (−2.25–(−0.45)
aBMD_HAZ_ Z-score TBLH	−0.74 ± 1.19 −0.70 (−1.40–0.02)	−0.7 ± 0.78 * −0.64 (−1.18–(−0.26)	−0.17 ± 0.97 −0.02 (−0.49–0.43)	−1.16 ± 1.03 * −1.31 (−1.79–(−0.4)
BMC TBLH [g]	691.99 ± 270.62 * 650.60 (514.78–784.82)	773.23 ± 185.83 731.28 (634.68–910.76)	1437.98 ± 516.97 1495.65 (969.82–1857.28)	1776.44 ± 2949.76 * 1332.80 (874.82–1642.41)

*, #, ^—values marked with the same symbols in the same row differ significantly from each other in terms of the given *p* value.

**Table 4:** The correlation between BMC Spine, aBMD Spine, BMAD, BMC TBLH, aBMD TBLH, Z-scores (before and after adjustment for height), calendar age, and individual auxological parameters and laboratory test results in the entire analyzed group of children hospitalized at the Department of Endocrinology and Metabolic Diseases of the PMMH-RI between 2020 and 2023 for various reasons (* *p* < 0.05).

	BMC Spine	aBMD Spine	BMAD	BMD for Age Z-Score Spine	BMD_HAZ_ Z-Score Spine	BMC TBLH	aBMD TBLH	BMD for Age Z-Score TBLH	BMD_HAZ_ Z-Score TBLH
Age [years]	0.727 *	0.684 *	0.506 *	−0.066	−0.169 *	0.389 *	0.796 *	−0.014	−0.15
HSDS	0.519 *	0.472 *	0.334 *	0.560 *	0.007	0.199 *	0.503 *	0.719 *	0.08
BMI SDS	0.431 *	0.470 *	0.450 *	0.558 *	0.348 *	0.106	0.461 *	0.638 *	0.43 *
Serum calcium	−0.228 *	−0.256 *	−0.24 *	−0.028	−0.013	−0.195 *	−0.222 *	0.001	0.07
Serum phosphate	−0.298 *	−0.283 *	−0.223 *	0.063	0.118	−0.259 *	−0.277 *	−0.008	0.04
Serum magnesium	−0.124	−0.144	−0.151	−0.084	−0.066	−0.083	−0.058	0.023	0.05
ALP	−0.377 *	−0.320	−0.242	0.137	0.091	−0.198 *	−0.228	0.307	0.26
Ca/Cr ratio	−0.196 *	−0.217 *	−0.199 *	−0.160	−0.117	−0.198 *	−0.238 *	−0.160	−0.11
PTH	−0.037	0.000	0.063	0.044	0.155	−0.066	−0.058	−0.030	0.11
25OHD	−0.208 *	−0.245 *	−0.228 *	−0.332 *	−0.189 *	−0.212 *	−0.265 *	−0.426 *	−0.18 *
Osteocalcin	−0.279 *	−0.300 *	−0.304 *	−0.023	−0.086	−0.214 *	−0.238 *	−0.041	−0.16
Crosslaps	−0.287 *	−0.334 *	−0.358 *	−0.094	−0.146	−0.197 *	−0.252 *	−0.124	−0.23 *
TSH	0.005	−0.032	−0.038	0.095	0.119	−0.063	−0.108	0.003	0.04
fT3	−0.151	−0.181 *	−0.194 *	0.042	−0.018	−0.118	−0.156	0.049	−0.02
fT4	−0.012	−0.031	−0.040	−0.080	−0.072	−0.058	−0.060	−0.069	−0.06
Estradiol (Total N = 86)	0.418 *	0.455 *	0.436 *	0.148	0.132	0.340 *	0.399 *	0.125	0.14
Estradiol (F = 55)	0.471 *	0.470 *	0.416 *	0.154	0.137	0.420 *	0.458 *	0.132	0.120
Estradiol (M = 31)	0.615 *	0.611 *	0.522 *	0.245	0.152	0.538 *	0.576 *	0.134	0.029
Testosterone (Total N = 97)	0.312 *	0.219 *	0.086	0.035	−0.055	0.340 *	0.351 *	0.029	−0.05
Testosterone (F = 46)	0.461 *	0.459 *	0.415 *	0.257	0.245	0.443 *	0.459 *	0.233	0.281
Testosterone (M = 51)	0.550 *	0.496 *	0.368 *	0.085	−0.077	0.526 *	0.585 *	0.184	0.051
IGF-1	0.577 *	0.563 *	0.439 *	0.325 *	0.088	0.608 *	0.662 *	0.423 *	0.18 *
IGF-1 SDS	0.039	0.063	0.097	0.436 *	0.288 *	0.063	0.077	0.492 *	0.43 *
IGF-BP3	0.474 *	0.476 *	0.397 *	0.311 *	0.211 *	0.471 *	0.525 *	0.401 *	0.30 *
Bone age	0.767 *	0.717 *	0.495 *	0.044	−0.114	−0.149	0.863 *	0.166	−0.05
Cortisol	−0.125	−0.083	−0.016	0.037	0.067	−0.149	−0.120	0.066	0.12

## Data Availability

Data are contained within the article.
